# ALICE: a randomized placebo-controlled phase II study evaluating atezolizumab combined with immunogenic chemotherapy in patients with metastatic triple-negative breast cancer

**DOI:** 10.1186/s12967-020-02424-7

**Published:** 2020-06-23

**Authors:** J. A. Kyte, A. Røssevold, R. S. Falk, B. Naume

**Affiliations:** 1 Department of Clinical Cancer Research, Oslo University Hospital, Oslo, Norway; 2 Department of Cancer Immunology, Oslo University Hospital, Oslo, Norway; 3 Department of Oncology, Oslo University Hospital, Oslo, Norway; 4 Oslo Centre for Biostatistics and Epidemiology, Oslo University Hospital, Oslo, Norway; 5 Institute of Clinical Medicine, University of Oslo, Oslo, Norway

**Keywords:** Breast cancer, Triple negative, Immunotherapy, Checkpoint inhibitor, Immunogenic cell death, PD-1, PD-L1, Anthracycline, Cyclophosphamide

## Abstract

**Background:**

Immunotherapy with checkpoint inhibitors (CI) represents an important novel development in cancer treatment. Metastatic triple-negative breast cancer (mTNBC) is incurable, with a median survival of only ~ 13 months. We have initiated the randomized placebo-controlled phase IIb study ALICE, evaluating PD-L1 blockade combined with immunogenic chemotherapy in mTNBC patients (n = 75). Intriguingly, the host immune response is strongly predictive for the effect of chemotherapy in mTNBC. In the ALICE trial, we release the brake on the immune response by use of atezolizumab, an inhibitory antibody against PD-L1. We utilize anthracyclines, shown to trigger the immune system, and low-dose cyclophosphamide, which has been reported to counter immunosuppressive cells.

**Methods:**

ALICE is a randomized, double-blind, placebo-controlled exploratory phase II study evaluating the safety and efficacy of atezolizumab when combined with immunogenic chemotherapy in subjects with mTNBC. The trial will enroll 75 evaluable subjects, randomized 2:3 into two arms (A:B). The patients receive identical chemotherapy, i.e. pegylated liposomal doxorubicin (PLD 20 mg/m^2^ intravenously every 2nd week) + cyclophosphamide (50 mg per day, first 2 weeks in each 4 week cycle). Patients in arm A receive placebo, while patients in arm B receive atezolizumab. The primary objectives are assessment of toxicity and progression-free survival. The secondary objectives include overall survival, tumor response rate, clinical benefit rate, patient reported outcomes, biomarkers and assessment of tumor-immune evolution during therapy.

**Discussion:**

The question of how CI should be combined with chemotherapy, is a key challenge facing the field. There is a strong preclinical rationale for exploring if anthracyclines, which are considered to induce immunogenic cell death, synergize with PD-L1 blockade, and if low-dose cyclophosphamide counters tumor tolerance. However, the data from patients is as yet very limited, and the clinical evaluation of these hypotheses is among the key objectives in the ALICE trial. The study includes extensive biobanking and translational sub-projects, also addressing other clinically important questions. These analyses may uncover mechanisms of drug efficacy or tumor resistance, and identify biomarkers allowing personalized therapy. If the trial suggests acceptable safety of the ALICE therapy and provide a signal of clinical efficacy, further studies are warranted.

*Trial registration* NCT03164993, May 24th 2017; https://clinicaltrials.gov/ct2/show/record/NCT03164993

## Background

The therapeutic options for metastatic triple-negative breast cancer (TNBC) are very limited. Interestingly, the host immune response is strongly predictive for the effect of chemotherapy in patients with TNBC [1]. In the present trial, we aim at releasing the brake on the immune response by the use of atezolizumab, an inhibitory antibody against Programmed Death Ligand-1 (PD-L1).

Immunotherapy with checkpoint inhibitors produces clinically important responses in several cancer forms, amid limited adverse effects [2–4]. This includes durable responses and improved survival in metastatic cancers. In 2019, immunotherapy was for the first time approved for use against breast cancer (BC), as atezolizumab was approved by the FDA and EMA for use in metastatic TNBC patients, based on the IMPASSION130 trial combining atezolizumab with nab-paclitaxel [5, 6]. This was the first randomized study demonstrating efficacy of a immunotherapy against TNBC. It should still be noted, that an effect was only found in the patients with PD-L1 expression up front, as measured by the SP142-assay. In the ALICE trial, we aim at triggering sensitivity to atezolizumab in patients that are otherwise not responsive, by use of selected chemotherapy, hypothesized to induce immunogenic cell death and counter immuno-suppressive cells.

There is compelling evidence from animal studies, supported by data from humans, that some chemotherapeutic agents are immunogenic [7–11]. Doxorubicin and cyclophosphamide have been suggested to be particularly powerful inducers of immunogenic cell death. Both agents fulfill 5/5 criteria established for assessing the immunogenicity of different chemotherapeutic drugs (Table 1 in [9]). There is also evidence from humans, particularly in breast cancer, indicating that the clinical effect of doxorubicin and cyclophosphamide depends on the host immune response [11]. Further, these agents have been shown to induce a Type I interferon immune response in breast cancer [8, 10]. In the present trial, we apply a low-dose, metronomic cyclophosphamide regime, that has been reported to counter immune suppression mediated by regulatory T cells (Tregs) and myeloid derived suppressor cells (MDSCs) [12]. Taken together, there is a strong rationale for synergy between the applied doxorubicin/cyclophosphamide regime and PD-L1 blockade [7].

PD-L1 is upregulated by IFNγ-related pathways, and the expression of PD-L1 is usually associated with immune activation. The fact that IMPASSION130 did not show an effect against PD-L1 negative TNBC, highlights the need to explore if more immunogenic chemotherapy, as employed in ALICE, can make immunologically “cold” tumours responsive to PD1/PDL1-blockade. Results from recent TNBC trials suggest that anthracyclines may be well suited for this purpose. The TONIC trial compared different regimes of induction therapy before PD1-blockade. Here, the group receiving doxorubicin recorded the highest response rate to nivolumab (anti-PD1), compared to other chemotherapy, radiation or no induction therapy [13]. There was, moreover, evidence of immune activation in the tumour after doxorubicin treatment. The Keynote 522 trial, which was conducted in the neoadjuvant setting, showed significantly increased response rates for the group receiving aPD1 as add-on to chemotherapy [14]. By contrast, the NEOTRIP trial did not show any benefit of anti PD-L1 (Gianni et al. SABCS December 2019). Interestingly. the chemotherapy regime in Keynote 522 contained anthracyclines and cyclophosphamide, while the NEOTRIP chemotherapy consisted only of taxanes. There are several possible explanations to these observations, and no conclusion can yet be drawn on which chemotherapy to combine with PD1-blokade. However, these clinical trial data, as well as the preclinical data mentioned above, suggests that the strategy of combining antracyclins with PD-L1 blockade is worth investigating, also in PD-L1 negative tumours.

## Methods

### Objectives

Primary objectives:

1. Assessment of toxicity of combined treatment with atezolizumab, pegylated liposomal doxorubicin and cyclophosphamide
2. Assessment of progression-free survival
  Secondary objectives:
3. Assessment of clinical response: Objective tumor response rate (ORR), duration of response (DR), durable tumor response rate (DRR; > 6 months), clinical benefit rate (CBR), overall survival (OS)
4. Assessment of changes in the immunological milieu in tumour and peripheral blood induced by the study therapy
5. Assessment of PD-L1 expression, mutation load and immune gene expression as biomarkers for clinical response
6. Assessment of patient reported outcomes, as measured by the Chalder Fatigue Questionnaire (FQ), an 11-point Numerical Rating Scale (NRS) for pain intensity and EORTC QLQ-C15-PAL
7. Assessment of immunological response
8. Identification of biomarkers for clinical response, toxicity and immune response
9. Characterization of tumor evolution induced by the study therapy
10. Characterization of changes in microbiota induced by the study therapy

### Study design

This is a randomized, double-blind, placebo-controlled exploratory phase II study evaluating the safety and efficacy of atezolizumab when combined with immunogenic chemotherapy in subjects with metastatic triple-negative breast cancer. Atezolizumab, pegylated liposomal doxorubicin and cyclophosphamide are the Investigational Medicinal Products (IMPs).

The patients (n = 75) will be randomized 2:3 into two arms (A:B):

- Arm A: Chemo (pegylated liposomal doxorubicin + cyclophosphamide) + placebo
- Arm B: Chemo (pegylated liposomal doxorubicin + cyclophosphamide) + atezolizumab

Upon radiographic disease progression per iRECIST, the patients must stop study treatment.

Patients that receive ≤ 3 doses of atezolizumab/placebo or ≤ 2 doses with PLD will not be considered evaluable per protocol, but will be included in the intention to treat (ITT) analysis.

To include a sufficient number of patients (75) in the per protocol (PP) analysis, the same number of patients will be added to study. The new subjects will be randomized according to the initial 2:3 ratio.

### Study treatment

- Atezolizumab (or placebo) will be administered intravenously 840 mg every 2nd week until disease progression or for a maximum of 24 months
- Chemotherapy will be administered as follows:
  - Pegylated liposomal doxorubicin 20 mg/m^2^ i.v. every 2nd week. An upper limit of 44 mg per dose will be applied to patients with a body surface area > 2.2 m^2^
  - Cyclophosphamide tablets 50 mg per day, daily as continuous treatment for the first 2 weeks in each 4 week period (i.e. every second 2 week cycle)
  - No upper limit for the number of cycles of pegylated liposomal doxorubicin/cyclophosphamide. Heart function will be monitored with regard to pegylated liposomal doxorubicin

### Rationale for chemotherapy regime

The chemotherapy regime used in the study is regarded as appropriate therapy for this patient group, without atezolizumab. The regime is expected to be well tolerated and applicable to most metastatic TNBC patients with ECOG 0-1, while also being sufficiently potent to suit those with an excellent performance status.

The chemotherapy regime and dosing schedule have been tailored to aid the effect of atezolizumab, which depends on immune effector cells for its activity. First, the chosen chemotherapeutic agents (anthracyclines and cyclophosphamide) are known to induce immunogenic cell death. Secondly, we apply the outlined dosage regime, rather than a high dose regime administered every 3rd/4th week, in order to maintain the leukocyte counts and the ability of the effector immune cells to respond. Anthracyclines are routinely administered at intervals ranging from one to four weeks in metastatic TNBC patients. Tregs and MDSCs represent important mediators of tumor tolerance and may oppose the effect of atezolizumab. The metronomic cyclophosphamide dosage chosen in the present study has been widely used to counter Tregs [12] and MDSCs and is also considered safe, as it has been combined with multiple other chemotherapeutic agents without causing important toxicity [15]. We include 14 day intervals without cyclophosphamide to allow for unsuppressed T cell proliferation and activity, which may be important for the atezolizumab effect.

We will use pegylated liposomal doxorubicin to minimize the adverse effects of anthracyclines on the heart and allow for continued treatment beyond the otherwise mandatory anthracycline limits. The possibility of long-term treatment is important in order to appropriately test checkpoint inhibitors like atezolizumab, as these drugs are known to induce durable responses in other patient groups. Pegylated liposomal doxorubicin is also administered without the need for corticosteroids, which is desirable for immunotherapy.

The standard pegylated liposomal doxorubicin dosage for breast cancer is 40–50 mg/m^2^ every 4th week. In Norway, the most widely used dose is 40 mg/m^2^. The dose chosen in our study is expected to be well tolerated, as the 40 mg/m^2^ is divided into two doses of 20 mg/m^2^ given every 2nd week. Some studies in breast cancer have used pegylated liposomal doxorubicin at 15–30 mg/m^2^ every 2nd week [15–19], or in combination with cyclophosphamide (500 mg/m^2^), and 5-fluorouracil (500 mg/m^2^) every 3rd week [20]. A dose of 20 mg/m^2^ has been well tolerated in combination with cyclophosphamide (50 mg/day) even in fragile, older patients [19] and is also tolerated by HIV positive patients with Kaposi sarcoma.

### Sample collection/biobanking

Samples are collected before, during and after therapy (time of progression/treatment discontinuation). Some of the biobanking procedures are not performed at all study centers. The following samples are collected: Biopsies, peripheral blood mononuclear cells (PBMC), plasma, serum, urine, feces and circulating tumor cells.

### Selected inclusion criteria

1. Metastatic or incurable locally advanced, histologically documented TNBC (absence of HER2, ER, and PR expression). ER and PR negativity are defined as < 1% and < 10%, respectively, of cells expressing hormonal receptors by IHC analysis
2. Adequate core or excisional study biopsy of a tumor lesion.
3. Measurable metastatic disease according to iRECIST
4. Eastern Cooperative Oncology Group (ECOG) performance status of 0 or 1
5. Signed Informed Consent Form
6. Women or men aged ≥ 18 years
7. A minimum of 12 months from adjuvant/neoadjuvant chemotherapy with anthracyclines to relapse of disease.
8. A maximum of one previous line of chemotherapy in the metastatic setting
9. Adequate organ function as defined in the protocol

### Selected exclusion criteria

1. Malignancies other than breast cancer within 5 years prior to randomization, with the exception of those with a negligible risk of metastasis or death and treated with expected curative outcome
2. Spinal cord compression not definitively treated with surgery and/or radiation, or previously diagnosed and treated spinal cord compression without evidence that disease has been clinically stable for > 2 weeks prior to randomization
3. Known CNS disease, except for treated asymptomatic CNS metastases, provided all of the following criteria are met:
  - Measurable disease outside the CNS
  - No metastases to mesencephalon, pons, medulla oblongata, or spinal cord
  - No evidence of progression after completion of CNS-directed therapy
  - No ongoing requirement for dexamethasone as therapy for CNS disease
  - No radiation of brain lesions within 14 days prior to randomization
  - No leptomeningeal disease
  - Patients with new asymptomatic CNS metastases detected at the screening scan must receive radiation therapy and/or surgery for CNS metastases. Following treatment, these patients may be eligible without the need for an additional brain scan prior to enrolment, if all other criteria are met.
4. Uncontrolled pleural effusion, pericardial effusion, or ascites.
5. Pregnant or breastfeeding
6. Received treatment with immune checkpoint modulators
7. Received treatment with systemic corticosteroids or other systemic immunosuppressive medications within 2 weeks prior to randomization, or anticipated requirement for systemic immunosuppressive medications during the trial
  - Patients who have received acute, low-dose, systemic immunosuppressant medications (e.g., a one-time dose of dexamethasone for nausea) may be enrolled in the study
  - Patients with a history of allergic reaction to IV contrast requiring steroid pre-treatment should have baseline and subsequent tumor assessments performed using MRI
  - The use of inhaled corticosteroids for chronic obstructive pulmonary disease, mineralocorticoids (e.g., fludrocortisone) for patients with orthostatic hypotension, and low-dose supplemental corticosteroids for adrenocortical insufficiency are allowed
8. Received anti-cancer therapy (medical agents or radiation) within 2 weeks prior to study Cycle 1, Day 1. Palliative radiotherapy for bone lesions is allowed up to 2 weeks before start of therapy.

### Outcome measures

#### Safety outcome measures

The safety outcome measures will be evaluated in the ITT population, as follows:

- Incidence, nature, and severity of adverse events graded according to NCI CTCAE v4.0
- Changes in vital signs, physical findings, and clinical laboratory results

#### Efficacy outcome measures

The PFS is defined as the time from randomization to the time of radiographic progression (as assessed by iRECIST) or death from any cause during the study. Data for patients with a PFS event who missed two or more assessments scheduled immediately prior to the date of the PFS event will be censored at the last tumor assessment prior to the missed visits. If no tumor assessment was performed after randomization, data will be censored at the date of randomization + 1 day. Clinical deterioration without objective radiological evidence will not be considered as documented disease progression. The primary efficacy outcome measure (PFS) is to be assessed in patients evaluable per protocol (PP).

The secondary efficacy outcome measures will be assessed in the PP population, ITT population and in the PD-L1-positive subpopulation.

### Statistics

A descriptive analysis of demographics, medical history, and clinical data will be performed.

The ITT population is defined as a full analysis set (FAS). The FAS is defined as all patients that have started therapy with at least one of the IMPs, and where data on the relevant endpoint is obtained. The safety will be evaluated in the ITT population.

The primary efficacy analyses will be performed on the patients that have completed at least 4 doses of atezolizumab/placebo and 3 doses of PLD (PP population). Secondary efficacy analyses will be performed on the PD-L1 positive subpopulation and on the ITT (FAS) population.

The primary efficacy analysis will be a descriptive analysis of progression free survival (PFS) in the atezolizumab arm (B), compared to the control arm (A). The HR for disease progression or death (arm B versus arm A) will be estimated using a Cox proportional hazards model. The confidence interval for the HR will be provided. Kaplan–Meier methodology will be used to estimate the median PFS for each treatment arm, and Kaplan–Meier curves will be produced.

Overall survival (OS) will be calculated from the time of randomization until death. Patients alive at the time of data analysis will be treated as censored. The HR for OS (arm B versus arm A) will be estimated using a Cox proportional hazards model. The CI for the HR will be provided. Kaplan–Meier methodology will be used to estimate the median OS for each treatment arm, and Kaplan–Meier curves will be produced.

Exploratory analyses will be carried out to evaluate the data of the immunological and molecular analyses (e.g. biomarker studies) carried out. The statistical analyses will be dependent on the biological factors investigated and the analysis methodology used, and will be defined separately for each molecular study.

We expect to reach the data-driven time point for PFS-analysis (90% PFS events in the PP population) about 6 months after inclusion of the last patient. If this is not met within 12 months after inclusion of the last patient, the PFS-analysis will be performed at this time point. The primary data analysis will be performed on the PP population. Further, the following factors will be studied:

- Tumor PD-L1 status
- Disease free interval: Less than 24 months versus > 24 months between end of adjuvant chemotherapy or surgery, whichever was last, and relapse. (Most TNBC patients receive adjuvant chemo for 6 months before/after radical surgery; a short disease-free interval suggests aggressive disease.)
- Prior chemotherapy against metastatic disease (no previous chemo versus previous chemo). Chemotherapy given in the neoadjuvant/adjuvant setting is not to be considered in this analysis
- Site(s) of metastases
- Molecular breast cancer profile and immune gene profile

## Discussion

### Study organization and timeline

Oslo University Hospital, Oslo, Norway, is the study sponsor. We have established 3 study centers in Norway (Oslo University Hospital, Stavanger University Hospital, St Olav University Hospital Trondheim) and two in Denmark Denmark (Rigshospitalet Copenhagen and Vejle University Hospital). The study opened in 2017, expanded to more sites in 2018 and has included 48 patients as of March 2020. We estimate a need to recruit 85 patients, to obtain the required 75 evaluable patients per protocol.

### Comments on study design and objectives

In the ALICE study, and a parallel trial in hormone receptor positive breast cancer (ICON; NCT03409198), we have chosen a randomized phase IIb design, with a limited number of patients. Historic controls are heterogeneous and of limited value. The key issue is that neither the effect nor the toxicity of adding a PD-L1 inhibitor to chemotherapy can be properly assessed in a one-armed study. On the other hand, a full-scale phase III trial, powered to show clinical efficacy with a *p* < 0.05, is not warranted, too resource demanding and ethically difficult, until a certain level of clinical data have been generated in TNBC.

The primary objective of the ALICE study is to assess the toxicity of atezolizumab as an add-on to chemotherapy, and provide leads on potential clinical efficacy against TNBC. Accordingly, the study was not powered to demonstrate a statistically significant (*p* < 0.05) clinical effect. If the study suggests acceptable toxicity and potential clinical benefit, a larger randomized study will be warranted.

Contrary to many phase II trials, the study includes a comprehensive assessment of quality of life (QoL). It is well known that the doctor´s perception and grading of side effects does not always correspond to the patient´s well-being. Further, the clinical efficacy in the palliative setting is certainly dependent on the effect on symptoms, not only survival time or objective tumour response. We aim to capture these aspects by use of a vailidated general form (EORTC QLQ-C15-PAL), tailored for metastatic patients, as well as specific read-outs for two key symptoms-fatigue and pain. The latter two symptoms were selected because they are considered to be important in this patient group.

Another key objective in ALICE is to assess the changes in the immunological milieu after study therapy. We will evaluate if immune activation is induced in the tumour by analysing repeated biopsies. Further, we will assess the effect of the study therapy at systemic level, by analyses of peripheral mononuclear blood cell, plasma and serum. Each of the study arms will be analysed separately, and compared.

The biomarker analyses represent another important objective in the study. These analyses, and other translational projects included in ALICE, may inform the selection of patient subgroups for later studies, identify strategies for improved therapy and allow for more personalized approaches. We aim to investigate mechanisms of effect and resistance, and assess the dynamic changes and tumor-immune co-evolution induced by the therapy. To this aim, consecutive biopsies, PBMCs and other samples are obtained.

Among the key challenges facing the field, is the question of which chemotherapy should be added to PD-L1/PD-1 blockers, and how the chemotherapy dosage should be tailored. In theALICE trial, we have in part based the choice of chemotherapy on current knowledge about immunogenic cell death and other effects of chemotherapy. Importantly, most of this knowledge is derived from preclinical models. There is sparse evidence from patients regarding the hypothesized beneficial effects of anthracyclines (immunogenic cell death) and low-dose cyclophosphamide (counter Tregs/MDSCs). A critical evaluation of these hypotheses, as included in the ALICE study, is required to inform the choice and dosing of chemotherapy for combination with checkpoint inhibitors in TNBC. The findings from ALICE may also be relevant to other cancer forms.

## Data Availability

Not applicable.

## Abbreviations

CBR: Clinical benefit rate
CI: Checkpoint inhibitor
CNS: Central nervous system
CR: Complete response
CTC: Circulating tumor cells
CTCAE: Common terminology criteria for adverse event
DOR: Duration of objective response
DR: Duration of response
DRR: Durable tumor response rate
ECOG: Eastern cooperative oncology group
EMA: European medicines agency
ER: Estrogen receptor
FAS: Full analysis set
HER2: Human epidermal growth factor receptor 2
HR: Hazard ratio
ICH: International conference on harmonization
IHC: Immunohistochemistry
IMP: Investigational medicinal product
ISH: In situ hybridization
ITT: Intention to treat
IV: Intravenous
MDSC: Myeloid-derived suppressor cells
ORR: Overall response rate
OS: Overall survival
PBMC: Peripheral blood mononuclear cell
PD: Progressive disease
PD-1: Programmed death 1
PD-L1: Programmed death ligand-1
PFS: Progression-free survival
PLD: Pegylated liposomal doxorubicin
PP: Per protocol
PR: Progesterone receptor
QLQ: Quality of life questionnaire
SD: Stable disease
TNBC: Triple-negative breast cancer
Tregs: T regulatory cells
